# Surgical Club of South West England

**Published:** 1986-02

**Authors:** 


### Surgical Club of South West England

*Meeting at Torquay on May 10th and 11th 1985*

### ASCENDING AORTO–BIFEMORAL GRAFTS

C. C. Wilmshurst, A. M. N. Gardner, K. M. Pagliero
Torbay Hospital, Torquay

Two cases were presented which posed particular problems with regard to aorto–bifemoral reconstruction.

One aged 65 had undergone previous abdominal surgery on three occasions and the other, aged 58, had a failed aorto–bifemoral graft.

Following repeat aortograms, it was decided to carry out ascending bifemoral reconstruction in order to bypass the peritoneal cavity as described by R. J. Baird.

In both cases, the femoral arteries were exposed first, to ascertain the adequacy of the run off. This being satisfactory, the ascending aorta was exposed through a median sternotomy. An 8 mm preformed axillo-bifemoral knitted Dacron Graft was pre-clotted and having applied a side occluding clamp to the ascending aorta, a small (1 cm) wedge was excised and the anastomosis carried out. The graft was tunnelled down in front of the posterior rectus sheath on the left side to the linea semilunaris where a small, vertical 2.5 cm incision was made through the rectus muscle so that each limb of the graft could be tunnelled to the femoral vesssels to complete the anastomoses. In one case, the route was over the inguinal ligament and in the other, an extraperitoneal tunnel was made beneath the inguinal ligament. Both cases are doing well at four years follow-up.

This approach is recommended in cases where an abdominal approach to the aorta may be hazardous, such as known peritoneal adhesions, previous multiple operations, incisional hernia and in cases of horseshoe kidney and previously failed aortic grafts.

### DOES DIPYRIDAMOLE AND ASPIRIN THERAPY PREVENT EARLY FAILURE OF FEMORODISTAL BYPASS GAFTS?

Charles A. Clyne
Torbay Hospital, Torquay

There is increasing evidence that anti-platelet regimes may prolong the life of prosthetic grafts but it may equally as well be important that antiplatelet regimes are commenced pre-operatively such that early platelet deposition on grafts is delayed and thus early occlusions may be limited.

Between July 1982 and July 1983, the 94 patients undergoing femoro-distal grafts for all grades of ischaemia were allocated (randomly) to treatment (PSAS) or control (C) groups. PSAS patients were given dipyridamole 200 mg b.d. and aspirin 300 mg daily for 6 weeks commencing 48 h pre-operatively, with dipyridamole 10 mg t.d.s. IV in the immediate post operative period. Graft (GS), limb (LS) and patient survival (PS) were followed for 1 year using clinical and Doppler assessment. Analysis was by life table (GS) and product limit (LS and PS). Probabilities (P) were calculated by the generalized Wilcoxon (Breslow) method. Fifty-two limbs received autogeneous vein (VG) and forty-two prosthetic grafts (PG).

| | GS (numbers) | | | |
|---|---|---|---|---|
| | Total | Fail | Patent | P |
| VG | | | | |
| C | 24 | 5 | 19 | 0.56 |
| PSAS | 28 | 8 | 20 | 0.56 |
| PG | | | | |
| C | 20 | 11 | 9 | 0.002 |
| PSAS | 22 | 3 | 19 | 0.002 |

| | LS (numbers) | | | |
|---|---|---|---|---|
| | Total | Amputation | Survive | P |
| VG | | | | |
| C | 24 | 2 | 22 | 0.29 |
| PSAS | 28 | 6 | 22 | 0.29 |
| PG | | | | |
| C | 20 | 7 | 13 | 0.10 |
| PSAS | 22 | 3 | 19 | 0.10 |

| | PS (numbers) | | | |
|---|---|---|---|---|
| | Total | Dead | Survive | P |
| VG+PG | | | | |
| C | 44 | 4 | 40 | 0.08 |
| PSAS | 50 | 10 | 40 | 0.08 |

The significant difference in prosthetic graft survival was achieved in the first post operative month which suggests that there is a place for the above antiplatelet regimen in the prevention of early PG occlusion.

### ALGODYSTROPHY

Mark Churcher
Plymouth General Hospital, Plymouth

This word is used to include both acute and chronic pain states ranging from acute causalgia to reflex sympathetic dystrophy.

Patients usually have a history of trauma, pain, evidence of abnormal sympathetic function and sensory changes with a sympathetic distribution pattern.

Thermography is a useful diagnostic tool, exemplified by some patients with post traumatic genital pain noted to have a cold thigh on the painful side.

Algodystrophy may follow aortic bifurcation surgery. A cool dysesthetic limb with a history of 'vascular pain' and fatigue after arterial surgery merits a trial sympathetic block before arteriography. The personal observation that sympathetic block relieves pain and reverses sensory changes following arterial dissection suggests that 'sympathetic afferents' in the arterial wall may initiate abnormal sympathetic reflex activity.

Psoas muscle injury or pressure ischaemia in pregnancy can produce a 'Psoas compartment syndrome'. Patients may present years later with a lower limb that tires easily and is both mildly dysesthetic and cool. Two patients presented with severe superficial dyspareunia in addition to other symptoms, had an appropriate history and physical signs and responded to sympathectomy.

Early sympathetic block cures many patients whilst treatment with Propranalol has proved more efficacious than Ketanserin. Long standing cases are difficult to help and some advocate sympathotomy rather than surgical sympathectomy in carefully selected patients.

### FOOT PUMP

A. M. N. Gardner
Torbay Hospital, Torquay

Phlebograms performed on my own foot done in the erect posture showing the capacious lateral plantar venous complex were presented. This venous pump is emptied by the action of weight bearing, but not by movement of the ankle. The pump can overcome a calf cuff inflated to 100 mm Hg. and dislocate a column of blood right up to the heart in the erect position; it is not dependent on muscular action because it still functions in paraplegics.

A pneumatic foot pad and impulse generator has been developed; it is designed to maintain the venous circulation in patients enclosed in plaster casts, who are unable to bear weight. This device now made by EBI Medical Systems Ltd., has proved highly successful in reducing the swelling after fractures and, in some cases, has avoided the necessity for fasciotomy. An unexpected benefit is marked reduction of pain that becomes obvious 1–2 hours after beginning the treatment.

### NEUROGENIC CLAUDICATION

D. S. Halpin
Torbay Hospital, Torquay and Princess Elizabeth Orthopaedic Hospital, Exeter

The differences in the symptoms between neurogenic and vascular claudication were discussed in some depth. The value of observing the claudication distance and the symptoms produced was stated. Caution in the interpretation of myelographic evidence of stenosis was emphasized strongly. The author's experience of operating on approximately 40 cases of neurogenic claudication were presented. Although the majority of patients had most satisfactory results, a good quarter of these patients continued to complain of some pain. As always the need to select patients very carefully for surgery was brought out. The talk finished with discussion of three individual cases, including one man who was putting up well with marked neurogenic claudication without treatment.

### USE OF DMSO IN THE TREATMENT OF INTERSTITIAL CYSTITIS

F. Parivar
Torbay Hospital, Torquay

Interstitial cystitis was first described by G. Hunner in 1914. Its symptoms include frequency and nocturia every ½–1 hour, urgency, suprapubic and perineal pain and sometimes haematuria. Its aetiology is basically unknown. There is growing evidence that the condition might be autoimmune in origin. The pathology is non-specific, however quantitative measurement of mast cells in the bladder muscle is thought to be diagnostic. Treatment is always initially conservative. Surgery is reserved as a last resort. One of the suggested methods of conservative treatment is the use of intravesical Dimethyl Sulfoxide (DMSO).

In a prospective study 13 patients with interstitial cystitis were selected for treatment. Diagnosis in this group of patients was made on the basis of history, cystoscopic findings and histology of the bladder mucosa. The patients were admitted to Day Beds every two weeks. They were catheterized and 50 mls. of 50% solution of DMSO was instilled in the bladder and the catheter removed. The solution was retained for 30 minutes and then drained by voiding. The treatment continued for six months. Three of the 13 patients dropped out because they 'were not getting any benefit'. All the remaining 10 patients had complete relief of their pain and significant immprovement in their frequency and nocturia. However bladder volume and cystoscopic appearances in these patients remained unchanged after the treatment. Overall, 5 of the patients were symptom free, 4 were significantly improved and 1 was moderately improved. It is therefore concluded that intravesical DMSO is a safe and cheap way of relieving the disturbing symptoms of interstitial cystitis.

### BENIGN BREAST DISEASE IN TORBAY

R. G. Hughes
Torbay Hospital, Torquay

In one year of consultant practice 132 patients with breast pathology or symptoms were seen, their ages range from 17–78 and 21 had cancer.

Of those with benign pathology, 70 had a lump and 60 had pain with or without a lump, 10 patients with cysts were seen and successfully treated by simple aspiration.

Approximately two thirds of patients presenting with breast pain were happy with reassurance that there was no underlying malignancy. The remainder were treated initially with Danazol, but only 2 of 14 obtained good symptom relief. Most of the other patients decided against further treatment, but one patient has run the gamut of Bromocriptine, Evening Primrose Oil, and Medroxyprogesterone with little improvement.

Discrete breast lumps were treated by trial aspiration to exclude cysts followed, usually, by excision. The diffusely lumpy breast was re-examined after six weeks and at suitable intervals thereafter unless there was cause for concern. Mammography was not often used and the commonest indication was for patient reassurance.

### POST-OPERATIVE CARE OF THE BREAST

David A. Griffiths
Yeovil District Hospital, Yeovil

The majority of breast operations in young women are for benign breast disease, e.g. fibroadenoma, cysts, abscesses and fistulae. The cosmetic result following surgery is often as important as the patient's relief at having the breast lesion removed. The careful use of circumareolar, submammary and axillary incisions improve the final appearance and frequently completely disguise the surgical incision. The normal erect position of the nipple is occasionally compromised following surgery, especially if the nipple has been explored and tight dressings applied. To reduce these problems a new nipple dressing was used following breast surgery.

Two hundred and thirty women at Yeovil District Hospital undergoing surgery for benign breast disease were treated with Stomahesive breast dressings. This wound dressing was a 3″ flat, circular sheet with a hole cut through the centre for the nipple. This was applied after surgery to the breast and used as the only dressing for seven to eight days. The women were encouraged to wear their brassieres immediately post-operatively to support the breast. The Stomahesive dressing adhered to the breast contours and the space left for the nipple encouraged normal healing.

The patients were sent home and examined in the Outpatients clinic within a few days. Removal of the nipple dressing was easy and all the wounds were completely healed without evidence of haematoma or infection.

The wounds were reassessed at one month post-operation and both the operated nipple and non-operated nipple were identical in shape and size.

In conclusion, the use of post-operative Stomahesive breast dressings gave satisfactory results for patient comfort, wound healing and cosmetic effect.

### 'THE OESOPHAGEAL LENGTHENING V–Y GASTROPLASTY WITH PARTIAL OR TOTAL FUNDOPLICATION FOR ACQUIRED SHORT OESOPHAGUS'

**K. Jeyasingham**
**Frenchay Hospital, Bristol**

One of the popular methods of surgically treating acquired short oesophagus with a dilatable stricture is to lengthen the oesophagus with a tube of stomach created from the lesser curve after the Collis gastroplasty fashion, and to then add a partial or total fundoplication as an anti-reflux measure. One of the technical problems with the procedure is the reduction in the size of the remaining fundus available for a fundoplication after the gastroplasty. The V–Y gastroplasty devised by the author, however, returns the neo-fundus to a normal size and contour. The tube of stomach is first created around a 44 FG bougie and the vertical gastric incision is now splayed in the form of an inverted V and sutured as the two limbs of the V meeting the vertical suture line on the neo-oesophagus to constitute an inverted Y. The partial or total fundoplication can now be easily completed using the neo-fundus and creating an intra-abdominal segment of 'oesophagus'.

Preliminary results on radiological, endoscopic, manometric and pH studies on approximately 26 patients who have undergone the procedure have been encouraging.

### LIBRARY SERVICES—A COMPUTER TERMINAL

**J. W. S. Rickett**
**Torbay Hospital, Torquay**

It is difficult to justify spending money on the library services when the clinical needs of a hospital are in direct competition for the capital or revenue.

A readership survey at Torbay Hospital Library was carried out to determine how often current journals are read. It was found that the major journals were regularly looked at, but it seemed hardly worthwhile taking the journals of the smaller specialties. The rising cost of binding was an added consideration and in many libraries the storage space is very limited. In recent times the photocopy service provided by the British Library is so good that any scientific paper can be to hand within 24 to 48 hours.

The library was faced with the dilemma of whether to increase the journal holding (at an average cost of £66 for each additional journal purchased) or consider other means of broadening the library services.

In 1983 the cost of a computer terminal was £670. This single payment provides on-line summaries in up to 50% of 3,000 journals from 70 countries over the past ten years. Surely this must be a better facility than adding 10 current journals? The computer terminal is small, quiet to run, quick in action and readily adaptable to the needs of the individual researcher.

The computer terminal is connected to Medline through the British Telecom service. The charges for an average search were £7.16 in 1983. Both the Library Committee and the District Treasurer thought that this was good value.

### MANAGEMENT OF THE BLADDER DURING AND AFTER ABDOMINAL SURGERY

**J. B. Bristol**
**Bristol Royal Infirmary, Bristol**

Perioperative catheterization is frequently performed during abdominal surgery. The principal indications are to debulk the pelvis, to monitor urine output, and occasionally for patient comfort or other reasons. The principal hazards of urethral catheterization are trauma, which may tend to stricture formation, and infection. Suprapubic catheterization may produce visceral damage or a vesico-cutaneous fistula. This latter route is not routinely used by surgeons operating within the abdomen. It does offer the theoretical advantages of permitting easy re-establishment of nominal micturition whilst sparing the urethra. A preliminary trial of operative supra-pubic catheterization was undertaken. 22 male patients, aged 53–84 years, judged to merit bladder catheterization at the time of surgery, were not catheterized urethrally at the beginning of the operation but suprapubically after opening the abdomen. A 16 F Argyle self-retaining catheter was inserted via a stab incision at the lower midline end of the wound or to one side of it. A hand behind the bladder protected the rectum. If the bladder was empty the procedure was undertaken at the end of the operation. The only operative complication was failure of insertion in one patient. The mean length of postoperative catheterization was 4.5 days. There were no urinary infections or other postoperative problems. Catheters were removed after spigotting had allowed normal micturition per urethram. A prospective study is now under way comparing the urethral and supra-pubic routes.

### POST ANAL REPAIR FOR IDIOPATHIC ANORECTAL INCONTINENCE

**Neil J. McC. Mortensen**
**Bristol Royal Infirmary, Bristol**

There can be no more distressing complaint than anorectal incontinence. Direct sphincter injuries following obstetric trauma or road traffic accidents may be repaired successfully by excision of scar tissue and an overlapping reconstruction of the external sphincter muscle. In the group of patients with so-called idiopathic anorectal incontinence no discrete sphincter defect can be identified. In these patients, often females aged 50 years and over, traction injury to the terminal branches of the pudendal nerves, perhaps during delivery or as a result of chronic straining at stool, may lead to neurogenic damage to the external sphincter and poor resting and voluntary contraction tone. Until recently such a situation becoming progressively distressing was often treated by colostomy.

The operation of post anal repair is indicated in patients with idiopathic anorectal incontinence after objective assessment with ano-rectal physiology investigations. After a limited bowel preparation the patient is placed in the lithotomy position with the buttocks strapped apart. A weak adrenaline saline solution is used to infiltrate the skin posterior to the anus in a broad V, also infiltrating more deeply in the plane between internal and external anal sphincter. The skin is incised in a similar broad V, and the anterior flap sewn back over the anus. The intersphincteric plane is developed, (a nerve stimulator may help to identify the external sphincter striated muscle) right up to the pelvic floor. Waldyer's fascia is then opened and the bowl shaped levator muscles identified. Pushing the anorectal angle forwards, sutures are placed approximating the puborectalis muscle and in turn the external sphincter. A redivac drain is placed in the space and the skin closed, often as a v Y–Y following indrawing of the anal canal by the procedure.

With a success rate of around 75% this simple procedure should be more widely offered to patients with anorectal incontinence.

